# Spacer rotation technique allows precise evaluation of gap balance in total knee arthroplasty

**DOI:** 10.1007/s00402-024-05253-1

**Published:** 2024-04-08

**Authors:** Georg Matziolis, Frank Layher, Sophia Vogt, Leah Bergner, Georgi Wassilew, Julia Kirschberg

**Affiliations:** 1 Orthopaedic Department, University Hospital Jena, Campus Eisenberg, Klosterlausnitzer Straße 81, 07607 Eisenberg, Germany; 2 Center for Orthopaedics, Trauma Surgery and Rehabilitation Medicine, University Medicine Greifswald, 17475 Greifswald, Germany

**Keywords:** Total knee arthroplasty, Spacer, Flexion gap, Extension gap, Symmetry, Asymmetry

## Abstract

The symmetry of the flexion and extension gap influences the functional and long-term outcome after total knee arthroplasty (TKA). Most surgeons check it by applying varus and valgus stress using spacers. This technique has limited accuracy and could be easily extended by rotational movement of the spacer. The objective was to determine the detection threshold and interobserver reliability of this technique. In an in vitro setting with a human cadaveric knee, gap asymmetries were simulated by different medially and laterally applied forces. Using an optical measurement system, the pivot point of the spacer was calculated as a function of the gap symmetry in the first part of the experiment. In the second part, the detection threshold and interobserver reliability of 4 surgeons were determined. For this purpose, gap asymmetries were adjusted to between 0 and 120N in a blinded trial. With a symmetrical gap, the centre of rotation of the spacer was located in the centre of the tibia. With increasing gap asymmetry, the centre of rotation of the spacer shifted to the tight side. This shift was approximately linearly dependent on the force difference. A perfectly balanced gap was detected by the examiners in 50% of the cases. From a force difference of 40N, all examiners identified the gap asymmetry in all cases (ICC = 1.0). The method of spacer rotation described is suitable for reliably detecting gap differences at ≥ 40N, independently of the examiner.

## Introduction

For a good clinical and long-term outcome after total knee arthroplasty (TKA), in addition to correct implant positioning, a balanced flexion and extension gap is particularly relevant [1].

There are various ways to check this: spacer blocks, laminar spreaders, tensioning devices, and force sensors [2]. All of these can be used with or without navigation or robotics. Although the different techniques lead to different results in gap determination and consequently in implant positioning, none has been shown to be superior [3–8].

With the introduction of electronic aids, it has been shown that even small gap asymmetries have a negative impact on clinical outcome [9–12]. For the Verasense sensor, pressure differences of less than 15 LBS were defined as the tolerance limit for balanced gaps [13, 14].

In studies with computer-assisted adjustment of the gaps (robotics and navigation), asymmetries of 1.5mm or 2mm already led to worse clinical outcomes [9, 15]. Computer-assisted adjustment of gap symmetry can be achieved with an accuracy of less than 1mm [16, 17].

However, most surgeons use spacers as conventional instruments to evaluate the gaps. It has been shown that surgeons cannot reproducibly balance a knee perfectly with conventional instruments and spacers [13, 18]. This may be due in part to the fact that spacers are inserted prior to component implantation, leading to a systemically error-prone evaluation of final stability. This has three causes:

1. In contrast to the original implant, there is no roll-back of the femur in flexion with the spacer, so that both the lateral collateral ligament (LCL) and the superficial medial collateral ligament (MCL) are more relaxed than after implantation of the prosthesis [19, 20].
2. In contrast to the spacer, the original inlay is not flat but has a posterior lip. The actual inlay height is therefore higher in flexion than in extension due to the roll-back of the femur [21].
3. The femoral component tightens the posterior capsule in extension through the posterior condyles and thus physiologically narrows the extension gap. This effect does not occur when the extension gap is determined with a spacer [16].

Trial implants are therefore better suited than spacers for determining the absolute gap height and thus the appropriate inlay height. However, the accuracy of gap symmetry determination with trial implants is inadequate [22].

Although spacers cannot accurately predict the absolute inlay height, gap symmetry is usually tested by applying varus and valgus stress with the spacer in place. However, this is subjective and opening of the gap in the millimetre range can visually only be estimated inaccurately. One limitation of spacers is the fact, that they are only able to evaluate the gap between femur and tibia in full extension and 90° of flexion. In contrast navigation and robotic systems can determine a gap envelope over the whole range of motion, when the knee is stressed manually or spring based tensioning devices are used.

A less examiner-dependent test could be advantageous here. For this purpose, the spacer could be inserted in extension or flexion and then rotated. In the case of gap symmetry, a central pivot point of the movement is to be expected. In the case of gap asymmetry, the spacer is expected to stick on the tighter side, the centre of rotation consequently shifting there.

The aim of the present study was to quantify the spacer rotation technique described and to test it with regard to detection threshold and interobserver reliability.

## Material and methods

**Fig. 1:**
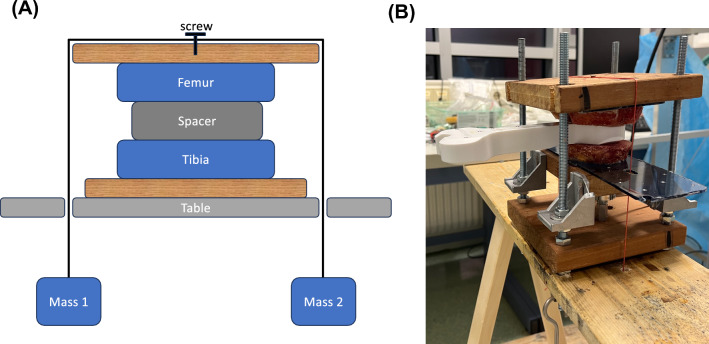
Schematic experimental setup (**A**) and photograph (**B**)

A human knee was freed from all soft tissues and a measured resection of the distal femur and proximal tibia was performed. The knee joint was loaded via pulleys with different weights for the medial and lateral compartments (Fig. 1A). The weights could be changed in a blinded manner for the examiners. In a preliminary trial, the construction was validated to ensure that the desired forces were indeed applied at the centre of the medial and lateral joint gap. For this purpose, two load cells were inserted into the joint gap and the forces were measured as a function of the weights.

The tests were performed with a force of 150N in one compartment (medial or lateral), as this corresponds to the average force of 160N in extension and 140N in flexion [23]. The force of the contralateral compartment was varied in steps between 30 and 150N, resulting in a force difference of 0–120N.

To determine the point of rotation of the spacer movement, both the spacer and the bones were marked with reflectors. These allowed an optical determination of their spatial position when performing the rotational movement of the spacer. This was done using a GOM Aramis (Zeiss, Germany), with a spatial resolution of 0.1mm. From successive frames, the trajectories of the optical markers and thus the position of the centre of rotation of the spacer could be calculated.

To determine interobserver reliability, the forces and sides (medial vs. lateral) were selected randomly and blinded to the examiner. The examiners inserted the spacer into the joint gap and assessed the pivot point on rotation of the spacer (medial/central/lateral) according to a medially tight, balanced or laterally tight gap (Fig. 1B). The trials were performed by 4 surgeons with surgical experience in knee arthroplasty.

### Statistical analysis

The pivot points of the spacer were graphically plotted on the tibia as a function of the force difference of the gaps. The percentage of correct determinations of the gap asymmetry by the examiners was determined as a function of the force difference of the gaps. From this, a cut-off value was determined for the smallest gap asymmetry that was still reliably determined by all examiners. The interobserver reliability was calculated using the ICC for forces below this cut-off value as well as above it. All tests were performed using SPSS (Ver. 24, IBM).

## Results

**Fig. 2:**
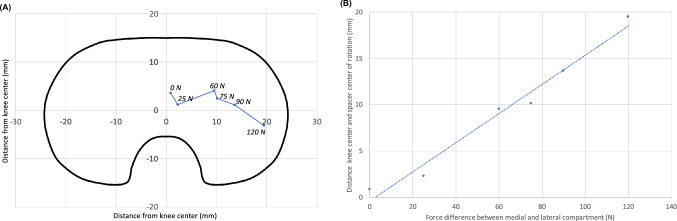
Centre of spacer rotation depending on extension gap asymmetry (0N … 120N) in projection to the tibia (**A**). The centre of spacer rotation moves towards the tight side with a approximately linear relationship between force and distance (**B**)

The centres of rotation of the spacer movement calculated from the videos are shown in Fig. 2A in projection onto the tibia. Whereas with a symmetrical gap the centre of rotation is in the middle of the tibia, increasing gap asymmetry (25N…120N) leads to a translation of the spacer centre of rotation to the edge of the tibia. The relationship between the mediolateral position of the spacer centre of rotation and the gap asymmetry is approximately linear (Fig. 2B).

**Fig. 3:**
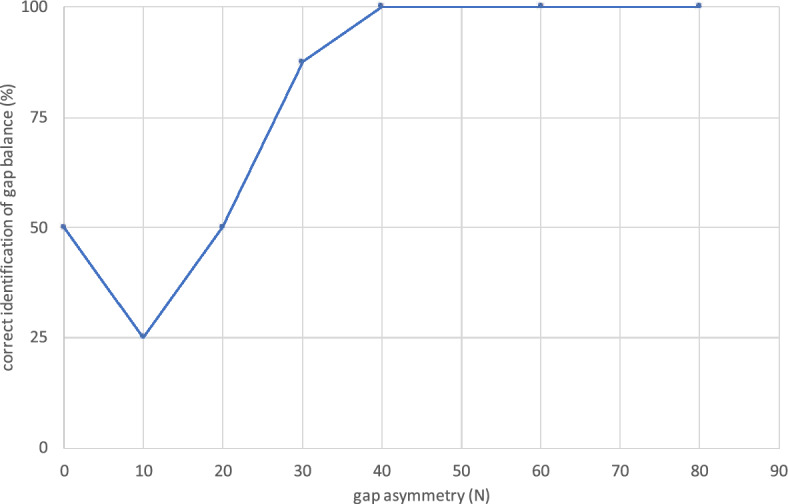
Correct identification of extension gap asymmetry depending on the force difference. A gap asymmetry of more than 30 N was detected by all observers

The proportion of gap asymmetry correctly estimated by the examiners using the spacer technique is shown in Fig. 3. A perfectly balanced gap was identified in 50% of the cases. Larger gap asymmetries were identified more frequently than smaller ones. The ICC was 0.561 for gap differences of less than 40N (moderate reliability) [24]. In contrast, a perfect match was found for a gap difference of 40N or more (ICC = 1). Thus, the method described is suitable for reliably detecting gap differences at ≥ 40N (= 9 LBS), independently of the examiner.

## Discussion

The main result of the present study is that the spacer rotation technique is suitable for detecting gap symmetry with sufficient precision in the context of total knee arthroplasty.

Spacers are usually inserted in such a way that varus and valgus stress is applied after insertion of the spacer and the respective lift of the femur from the spacer is visually estimated [25]. With navigation systems, an accurate result can be achieved that corresponds to the gap symmetry with trials [16].

The applied force during varus/valgus stress is subjective and cannot be objectified without the use of equipment. In addition, the minimal lift of the femur from the spacer can only be estimated inaccurately without a navigation system or robot and is therefore subject to error. Since the gap asymmetry is calculated as the difference between the medial and lateral lift-off, it is the result of two estimates, each of which is subject to error, and is therefore even less accurate according to the rules of error propagation.

An alternative technique is to estimate the varus and valgus stress force required to lift the femur off the spacer after insertion of the spacer. If the force required differs, there is a gap asymmetry. This requires the surgeon's ability to apply force in a reproducible manner. However, even with an identical direction of force, this is only possible with insufficient accuracy; with different directions of force (varus/valgus stress), an even greater scatter can be assumed.

The principle of the spacer rotation technique requires the translation of the spacer tray and observation of the resulting pivot point. The level of the translation force is irrelevant, the surgeon only being responsible for identifying the pivot point (medial/central/lateral). The present study shows that the mediolateral position of this pivot point is approximately linearly dependent on the gap asymmetry. The detection of a non-central pivot point is surprisingly accurate—possibly due to the possibility of multiple rapid repetition of the test. Force differences of 40N (= 9 LBS) could already be reliably detected by different surgeons (ICC = 1.0). This difference is well below the repeatedly published gap difference of 15 LBS, which a balanced gap should not exceed [13, 14]. Thus, in contrast to the classical use of spacers, the technique described is suitable for detecting a balanced gap with a high degree of certainty, even without electronic aids.

The main limitation of the study is the small number of cases, with only one cadaveric knee and the artificial creation of asymmetry through external force application without a collateral ligament apparatus and capsule. Different cadaver joints may have different static friction with different bone density [26]. However, an influence on the correlation shown between gap symmetry and pivot point of the spacer seems unlikely. The external application of force to simulate gap asymmetry has advantages and disadvantages. On the one hand, it allowed a reproducible and finely set adjustment of the gap asymmetry. On the other hand, weights exert constant forces on the medial and lateral compartment. This only reflects reality to a limited extent, as ligament and capsule have non-linear force/strain curves [23, 27]. However, these differences would only occur at significantly higher forces than clinically relevant gap asymmetry and are therefore irrelevant for the question investigated here. Another limitation of the study is, that all soft tissue was stripped off the bone and external load was used to simulate the tension of the medial and lateral stabilizing structures. Given, that no external load was applied centrally, the posterior cruciate ligament was not simulated, so that the results can only be transferred to posterior cruciate sacrificing surgical technique. Another limitation is the measurement of only the extension gap. The difference of the distal and posterior bone cuts of the femur is only minor and medial/lateral symmetric, so that similar results are expected for the flexion gap.

In conclusion, the spacer rotation technique allows the examiner-independent detection of gap symmetry at ≥ 40N (= 9 LBS), with an accuracy that is in the range of more sophisticated electronic tools.

## Data Availability

Data supporting Figs. 2 and 3 are available on request.

## References

[1] Mihalko WM, Saleh KJ, Krackow KA, Whiteside LA (2009). Soft-tissue balancing during total knee arthroplasty in the varus knee. J Am Acad Orthop Surg.

[2] D’Lima DD, Colwell CW (2017). Intraoperative measurements and tools to assess stability. J Am Acad Orthop Surg.

[3] D’Elicio DG, Attanasio M, Ruffo G (2021). Improving radiographic patello-femoral tracking in total knee arthroplasty with the use of a flexion spacer: a case-control study. Knee Surg Sports Traumatol Arthrosc Off J ESSKA.

[4] In Y, Kim S-J, Kim J-M (2009). Agreements between different methods of gap balance estimation in cruciate-retaining total knee arthroplasty. Knee Surg Sports Traumatol Arthrosc Off J ESSKA.

[5] Murphy GT, Shatrov J, Duong J, Fritsch BA (2023). How does the use of quantified gap-balancing affect component positioning and limb alignment in robotic total knee arthroplasty using functional alignment philosophy? A comparison of two robotic platforms. Int Orthop.

[6] Sava M-P, Hara H, Alexandra L (2023). Verasense sensor-assisted total knee arthroplasty showed no difference in range of motion, reoperation rate or functional outcomes when compared to manually balanced total knee arthroplasty: a systematic review. Knee Surg Sports Traumatol Arthrosc Off J ESSKA.

[7] Shatrov J, Murphy GT, Duong J, Fritsch B (2021). Robotic-assisted total knee arthroplasty with the OMNIBot platform: a review of the principles of use and outcomes. Arch Orthop Trauma Surg.

[8] ten Ham AM, Heesterbeek PJC, van der Schaaf DB (2013). Flexion and extension laxity after medial, mobile-bearing unicompartmental knee arthroplasty: a comparison between a spacer- and a tension-guided technique. Knee Surg Sports Traumatol Arthrosc Off J ESSKA.

[9] Chia Z-Y, Pang H-N, Tan M-H, Yeo S-J (2018). Gap difference in navigated TKA: a measure of the imbalanced flexion-extension gap. SICOT-J.

[10] Gustke KA, Golladay GJ, Roche MW (2014). Primary TKA patients with quantifiably balanced soft-tissue achieve significant clinical gains sooner than unbalanced patients. Adv Orthop.

[11] Meneghini RM, Ziemba-Davis MM, Lovro LR (2016). Can intraoperative sensors determine the “Target” ligament balance? Early outcomes in total knee arthroplasty. J Arthroplasty.

[12] Sun C, Zhao Z, Lee WG (2022). Sensor-guided gap balance versus manual gap balance in primary total knee arthroplasty: a meta-analysis. J Orthop Surg.

[13] Golladay GJ, Bradbury TL, Gordon AC (2019). Are patients more satisfied with a balanced total knee arthroplasty?. J Arthroplasty.

[14] Gustke KA, Golladay GJ, Roche MW (2014). A new method for defining balance: promising short-term clinical outcomes of sensor-guided TKA. J Arthroplasty.

[15] Keggi JM, Wakelin EA, Koenig JA (2021). Impact of intra-operative predictive ligament balance on post-operative balance and patient outcome in TKA: a prospective multicenter study. Arch Orthop Trauma Surg.

[16] Jhurani A, Agarwal P, Aswal M (2020). Do spacer blocks accurately estimate deformity correction and gap balance in total knee arthroplasty? A prospective study with computer navigation. Knee.

[17] Stiehl JB, Heck DA (2015). How precise is computer-navigated gap assessment in TKA?. Clin Orthop.

[18] Elmallah RK, Mistry JB, Cherian JJ (2016). Can we really “Feel” a balanced total knee arthroplasty?. J Arthroplasty.

[19] Kinsey TL, Mahoney OM (2018). Balanced flexion and extension gaps are not always of equal size. J Arthroplasty.

[20] Nitta A, Wada K, Hamada D (2023). Insertion of a spacer block translates the tibia anteriorly during evaluation of soft tissue balance in cruciate-retaining total knee arthroplasty. Knee.

[21] Matsui Y, Matsuura M, Hidaka N (2021). A tensor with a flat surface overestimates midflexion laxity in total knee arthroplasty: Comparison between a tensor with a flat-shaped surface and a tensor with an insert-shaped surface. Knee.

[22] MacDessi SJ, Gharaibeh MA, Harris IA (2019). How accurately can soft tissue balance be determined in total knee arthroplasty?. J Arthroplasty.

[23] Oshima Y, Iizawa N, Takai S, Majima T (2021). Optimal distraction force for evaluating tibiofemoral joint gaps in posterior stabilized total knee arthroplasty. J Nippon Med Sch Nippon Ika Daigaku Zasshi.

[24] Portney LG, Watkins MP (2009). Foundations of clinical research: applications to practice.

[25] Lee H-J, Kim SH, Park Y-B (2020). Selective medial release using multiple needle puncturing with a spacer block in situ for correcting severe varus deformity during total knee arthroplasty. Arch Orthop Trauma Surg.

[26] Orlando F, Arosio F, Arosio P, Di Stefano DA (2019). Bone density and implant primary stability. A study on equine bone blocks. Dent J.

[27] Nagai K, Muratsu H, Matsumoto T (2014). Soft tissue balance changes depending on joint distraction force in total knee arthroplasty. J Arthroplasty.

